# Assessing genotype-phenotype associations in three dorsal colour morphs in the meadow spittlebug *Philaenus spumarius* (L.) (Hemiptera: Aphrophoridae) using genomic and transcriptomic resources

**DOI:** 10.1186/s12863-016-0455-5

**Published:** 2016-11-15

**Authors:** Ana S. B. Rodrigues, Sara E. Silva, Francisco Pina-Martins, João Loureiro, Mariana Castro, Karim Gharbi, Kevin P. Johnson, Christopher H. Dietrich, Paulo A. V. Borges, José A. Quartau, Chris D. Jiggins, Octávio S. Paulo, Sofia G. Seabra

**Affiliations:** 1 Computational Biology and Population Genomics Group, cE3c – Centre for Ecology, Evolution and Environmental Changes, Departamento de Biologia Animal, Faculdade de Ciências da Universidade de Lisboa, Campo Grande, Lisbon, P-1749-016 Portugal; 2 Centro de Estudos do Ambiente e do Mar (CESAM), DBA/FCUL, Lisbon, Portugal; 3 Centre for Functional Ecology, Department of Life Sciences, University of Coimbra, Coimbra, Portugal; 4 Edinburgh Genomics, Ashworth Laboratories, King’s Buildings, The University of Edinburgh, Edinburgh, EH9 3JT UK; 5 Illinois Natural History Survey, Prairie Research Institute, University of Illinois, Champaign, IL USA; 6 cE3c – Centre for Ecology, Evolution and Environmental Changes/Azorean Biodiversity Group and Universidade dos Açores – Departamento de Ciências e Engenharia do Ambiente, Angra do Heroísmo, Açores Portugal; 7 Department of Zoology, University of Cambridge, Downing Street, Cambridge, CB2 3EJ UK

**Keywords:** Association study, Colour polymorphism, *de novo* genome assembly, *de novo* transcriptome assembly, Meadow spittlebug

## Abstract

**Background:**

Colour polymorphisms are common among animal species. When combined with genetic and ecological data, these polymorphisms can be excellent systems in which to understand adaptation and the molecular changes underlying phenotypic evolution. The meadow spittlebug, *Philaenus spumarius* (L.) (Hemiptera, Aphrophoridae), a widespread insect species in the Holarctic region, exhibits a striking dorsal colour/pattern balanced polymorphism. Although experimental crosses have revealed the Mendelian inheritance of this trait, its genetic basis remains unknown. In this study we aimed to identify candidate genomic regions associated with the colour balanced polymorphism in this species.

**Results:**

By using restriction site-associated DNA (RAD) sequencing we were able to obtain a set of 1,837 markers across 33 individuals to test for associations with three dorsal colour phenotypes (*typicus*, *marginellus*, and *trilineatus*). Single and multi-association analyses identified a total of 60 SNPs associated with dorsal colour morphs. The genome size of *P. spumarius* was estimated by flow cytometry, revealing a 5.3 Gb genome, amongst the largest found in insects. A partial genome assembly, representing 24% of the total size, and an 81.4 Mb transcriptome, were also obtained. From the SNPs found to be associated with colour, 35% aligned to the genome and 10% to the transcriptome. Our data suggested that major loci, consisting of multi-genomic regions, may be involved in dorsal colour variation among the three dorsal colour morphs analysed. However, no homology was found between the associated loci and candidate genes known to be responsible for coloration pattern in other insect species. The associated markers showed stronger differentiation of the *trilineatus* colour phenotype, which has been shown previously to be more differentiated in several life-history and physiological characteristics as well. It is possible that colour variation and these traits are linked in a complex genetic architecture.

**Conclusions:**

The loci detected to have an association with colour and the genomic and transcriptomic resources developed here constitute a basis for further research on the genetic basis of colour pattern in the meadow spittlebug *P. spumarius*.

**Electronic supplementary material:**

The online version of this article (doi:10.1186/s12863-016-0455-5) contains supplementary material, which is available to authorized users.

## Background

Understanding the genetic basis underlying phenotypic variation responsible for evolutionary change and adaptation in natural populations remains a major goal and one of the most interesting challenges in evolutionary biology. Not long ago, despite the development of new molecular tools, establishing genotype-phenotype associations, mapping adaptive loci, and identifying gene function, was limited to a few *taxa* due to technological and cost constraints. With the latest advances in sequencing technologies, the relationships between genetic variation and adaptive traits can now be investigated in a broader range of species for which, in some cases, there is extensive knowledge of ecological and evolutionary history, but few genomic resources [1–7]. Moreover, with the development of population genomics it has become possible not only to assess the genetic basis of adaptation directly at a genomic level, but also to distinguish the evolutionary effects of forces acting on the whole genome from those influencing only particular loci [8, 9].

Intraspecific colour variation is commonly found in many different *taxa*, including mammals [10], fishes [11], amphibians [12], reptiles [13, 14], birds [15, 16], and many invertebrates (e.g. land snails, spiders, grasshoppers and butterflies; see [17] for references). Colour patterns may serve a wide variety of adaptive functions, ranging from a visual signal used in mate choice, to crypsis or aposematism to avoid predators, to aiding in the regulation of body temperature [18]. Through their interactions with other physiological and/or ecological traits, colour polymorphisms may also influence the habitat choice, dispersal capability and adaptation to a changing or novel environment, thus influencing the ecological success and evolutionary dynamics of populations and species [19]. When combined with genomic and ecological data, these colour polymorphisms can be an excellent system for understanding adaptation and speciation and for the study of the micro-evolutionary forces that maintain genetic variation [20]. Negative frequency-dependent selection, resulting from processes such as predation or sexual selection [21–23], heterozygote advantage [24], and disruptive selection/divergence with gene-flow [25, 26] are some of the mechanisms suggested to be involved in the maintenance of colour polymorphisms. Alternative strategies that result in almost the same fitness values for colour morphs have also been reported [27].

The meadow spittlebug, *Philaenus spumarius* (Linnaeus, 1758) (Hemiptera, Aphrophoridae), a widespread and highly polyphagous sap-sucking insect species in the Holarctic region, shows a well studied balanced polymorphism of dorsal colour/pattern variation [28]. It is the most investigated species of its genus and has high genetic and morphological variation [29]. Sixteen adult colour phenotypes are known to occur in natural populations [30] but only 13 are referred in the literature. These are divided into non-melanic (*populi*, *typicus*, *vittatus*, *trilineatus* and *praeustus*) and melanic forms (*marginellus*, *flavicollis*, *gibbus*, *leucocephalus*, *lateralis*, *quadrimaculatus*, *albomaculatus* and *leucopthalmus*) [28, 30–32]. The occurrence and frequency of the colour phenotypes differ among populations and may result from different selective pressures such as habitat composition, climatic conditions (including altitudinal and latitudinal gradients), industrial melanism and predation (reviewed in [30, 32]). Silva and colleagues [33] have shown higher longevity and fertility of the *trilineatus* phenotype in laboratory conditions, which was also found to have the highest reflectance [34] and to be more prone to parasitoid attacks [35], supporting the idea that complex mechanisms are involved in the maintenance of this polymorphism. Crossing experiments have revealed the Mendelian inheritance of this trait, which is mainly controlled by an autosomal locus *p* with seven alleles, with complex dominance and co-dominance relationships, being likely regulated by other loci [31, 36]. The *typicus* phenotype is the most common (over 90% frequency in most populations) and it is the bottom double recessive form. It is believed to be the ancestral form because its main colour pattern characteristics are shared with several other cercopid species [36]. The completely melanic form *leucopthalmus* is dominant over *typicus*, and several other forms, with pale heads and/or spots, are dominant over the completely dark form. The *trilineatus* phenotype, pale with three dark stripes, is controlled by the top dominant allele *p* ^T^ [36, 37]. Halkka and Lallukka [38] suggested the colour genes may be linked to genes involved in response to the physical environment through epistatic interactions, constituting a supergene, and selection may not be directly related to colour. Evidence that balanced polymorphisms can result from tight genetic linkage between multiple functional loci, known as supergenes [39], has been reported in mimetic butterflies [40, 41], land snails [42] and birds [43]. In *P. spumarius* the genetic architecture of its balanced dorsal colour polymorphism and the possible existence of a supergene remain to be investigated.

A genome-wide association study has the potential to identify the genetic and/or genomic region(s) associated with these dorsal colour patterns. In this study we used restriction site-associated DNA (RAD) sequencing [1] to obtain a set of Single Nucleotide Polymorphisms (SNPs) that were tested for associations with three dorsal colour phenotypes in *P. spumarius*. The phenotypes used were: *typicus* (TYP), the most common and non-melanic recessive phenotype; *trilineatus* (TRI), the non-melanic dominant phenotype; and *marginellus* (MAR), the most common melanic phenotype found in the population from which samples were collected. The first partial draft genome and transcriptome of *P. spumarius* are presented here and were used to help the characterisation of the genomic regions found to be associated with colour variation. The size of the genome of this insect species was also estimated by flow cytometry.

## Methods

This research does not involve any endangered or protected species and did not require any permits to obtain the spittlebug individuals.

### Sampling and DNA extraction

A total of 36 female specimens of *P. spumarius* from three different colour phenotypes – 12 *typicus* (TYP), 12 *trilineatus* (TRI), and 12 *marginellus* (MAR) – were collected from a Portuguese population near Foz do Arelho locality (39°25'2.95"N; 9°13'39.18"W) in 2011. Adult insects were captured using a sweep net suitable for low-growing vegetation and an entomological aspirator (pooter). Specimens were preserved in absolute ethanol and stored at 4°C. The wings and abdomen were removed to avoid DNA contamination by endosymbionts, parasitoids and parasites and only the thorax and head were used. Genomic DNA was extracted using the DNeasy Blood & Tissue Kit (Qiagen).

### Illumina sequencing of genomic libraries

Three RAD libraries with twelve individuals each were prepared following a modified RAD sequencing protocol [1], using PstI-HF (New England BioLabs) restriction enzyme to digest 300 ng of genomic DNA per sample. Digested DNA was ligated to P1 barcoded adapters using twelve different barcodes for each library. Adapter-ligated fragments were pooled and sheared targeting a 500 bp average fragment size using a sonicator. To remove adapter dimers, libraries were purified with Agencourt AMPure XP (Beckman Coulter) magnetic beads after P2 adapter ligation with a volume DNA/beads ratio of 1:0.8. After end-repair using a commercial kit (New England BioLab), libraries were amplified by Polymerase Chain Reaction (PCR) performing an initial denaturation step at 98°C for 30 s, followed by 18 cycles of one denaturation step at 98°C for 10 s, annealing at 65°C for 30 s, extension at 72°C for 30 s and a final 5 min extension step. PCR-enriched libraries were purified with AMPure XP beads and the DNA concentration of each library was quantified in a Qubit 2.0 (Invitrogen). Libraries, in a proportional representation, were paired end sequenced in three lanes of an Illumina HiSeq 2000 at Genepool (Ashworth Laboratories).

### SNP calling and genotyping

Raw reads were trimmed, demultiplexed and aligned using the pyRAD software pipeline v3.0.5 [44], which follows the method of [45]. Reads were first clustered by individual and highly similar reads assembled into “clusters” using the programs MUSCLE v3.8.31 [46] and VSEARCH v1.9.3 [47] that allowed reads within “clusters” to vary not only for nucleotide polymorphisms but also for indels. All bases with a Phred quality score below 20 were converted to N (undetermined base). For each individual, consensus sequences based on estimates of the sequencing error-rate and heterozygosity were obtained for each locus. Similarity threshold required to cluster reads together and individuals into a locus was 0.88. Minimum “cluster” depth for each individual was six reads. Only loci with a minimum coverage of nine individuals (25%) were retained in the final dataset. To limit the risk of including paralogs in analysis, loci sharing more than 50% heterozygous sites were not considered and the maximum number of heterozygous sites in a consensus sequence (locus) allowed was five. After clustering sequences, a data matrix for each locus was generated. Further filtering and summary statistics were, posteriorly, performed using VCF Tools v 0.1.13 [48]. Loci were excluded from the final matrix based on (i) a missing data higher than 90% per individual, (ii) a minor allele frequency lower than 5% and (iii) a missing data per loci higher than 25%. Linkage disequilibrium (LD) was also measured using the squared correlation coefficient (*r* ^2^) in VCFtools. In association analysis, the detection of statistical associations may be affected when a marker is replaced with a highly correlated one [49]. Taking this into account, highly correlated SNPs in the same locus (*r* ^2^ = 1) were randomly eliminated and only one of them was retained in the final VCF matrix. The filtered VCF file with the genotypes for each individual was converted into the file formats needed for further analyses using PGDSpider v 2.0.4.0 [50], fcGENE v1.0.7 [51] and/or using customised python scripts.

### Association with dorsal colour phenotypes

For the SNPs dataset, single-SNP associations between allele frequencies and dorsal colour phenotypes were tested using a Fisher’s exact test of allelic association in PLINK v 1.07 [52]. Three pairwise analyses were performed: MAR vs. TRI, MAR vs. TYP and TRI vs. TYP. Allele frequencies in each pair, the odds ratio and *p-*values were obtained for each SNP and a false discovery rate (FDR) of 5% was applied [53] to each pairwise analysis to test for false positives.

To test for single and multi-SNP correlations between SNPs and colour morphs, a Bayesian Variable Selection Regression (BVSR) model proposed by [54] was also performed in the same three pairs and carried out in piMASS v 0.9. Generally used for association studies with continuous response variables, piMASS is also appropriate for studies with binary phenotypes [54]. This method uses the phenotype as the response variable and genetic variants (SNPs) as covariates to evaluate SNPs that may be associated with a particular phenotype [54]. SNPs statistically associated with phenotypic variation are identified by the posterior distribution of γ, or the posterior inclusion probability (PIP). In our multi-locus analyses, markers with a PIP greater than 99% empirical quantile (PIP_0.99_ SNPs) were considered as highly associated with colour morphs. For all PIP_0.99_ SNPs we reported their PIP and the estimates of their phenotypic effect (β). A positive β in the pairwise morph1-morph2 (e.g. MAR-TRI) analysis means that the frequency of the minor allele (maf) is higher in morph2 (TRI in the example) and a negative β means that maf is higher in morph1 (MAR in the example). Thus, to investigate the phenotypic effect size of each PIP_0.99_ SNP, the | β | was considered. The model contains additional parameters that are estimated from the data: proportion of variance explained by the SNPs (PVE), the number of SNPs in the regression model (nSNPs) and the average phenotypic effect of a SNP that is in the model (σSNP). For all pairwise analyses, we obtained 4 million Markov Chain Monte Carlo samples from the joint posterior probability distribution of model parameters (recording values every 400 iterations) and discarded the first 100,000 samples as burn-in. piMASS also outperforms a single-SNP approach to detect causal SNPs even in the absence of interactions between them [54]. For single-marker tests, SNPs above 95% empirical quantile for Bayes Factor (BF) (BF_0.95_ SNPs) were considered to be strongly associated to the colour phenotypes. Those above 99% empirical quantile for BF (BF_0.99_ SNPs) were considered to have the strongest associations. Imputation of the missing genotypes was performed in BIMBAM v1.0 [55].

Genetic differences among populations were tested using a *G*–test [56] and estimates of *F*_*ST*_ were obtained following the method of [57] implemented in GENEPOP v4.2.2 [58]. To better visualise and explore the correlation between significant SNPs, obtained in the several association analyses, and colour phenotypes, a Principal Component Analysis (PCA) was done using R Package SNPRelate (Bioconductor v3.2; R v3.2.3) implemented in the vcf2PCA.R script [59].

### *De novo* sequencing and assembly of the meadow spittlebug genome

To attempt potential *de novo* assembly of the genome, genomic DNA of one *P. spumarius* individual from Quinta do Bom Sucesso, Lagoa de Óbidos (Portugal) was extracted using the DNeasy Blood & Tissue Kit (Qiagen) and sequenced externally in GenoScreen (Lille, France) (http://www.genoscreen.fr/). A whole-genome shotgun sequencing approach using one lane of Illumina HiSeq 2000 to generate a paired-end library of approximately 366 million 100 bp reads was carried out. After sequencing, the quality of the sequence reads was assessed in FastQC v0.10.1 [60] and low quality sequences were trimmed by using Trimmomatic v 0.35 [61] and the default parameters. *De novo* assembly of large genomes tends to be computationally demanding, requiring very large amounts of memory to facilitate successful assembly. Taking these conditions into account, the assembler SOAPdenovo2 [62, 63] was chosen to assemble the sequenced *P. spumarius* genome. This assembler implements the *de Bruijn* graph algorithm tailored specifically to perform the assembly of short Illumina sequences and is optimised for large genomes. A k-mer parameter of 33 was used for this assembly. The quality of the assembly results was investigated through several metrics: N50, percentage of gaps, number of *contigs*, number of scaffolds and genome coverage (total number of base pairs).

### *De novo* sequencing and assembly of the meadow spittlebug transcriptome

Fresh adult specimens of *P. spumarius* were obtained from Lexington, Fayette Co., Kentucky, USA in July 2013 and frozen at −80°C. Total RNA was extracted from 6 adult specimens by first grinding the entire body using a 1 mL glass tissue grinder with 1 mL Trizol (Invitrogen). This was followed by passing the homogenate over a Qiagen Qiashredder column. The eluate was extracted with 200 μL chloroform, and the RNA was precipitated with 500 μL isopropanol. Pellets were resuspended in RNAse-free water.

Paired-end RNA libraries were prepared using Illumina’s TruSeq Stranded RNA sample preparation kit with an average cDNA size of 250 bp (range 80–550 bp). These libraries were sequenced using an Illumina HiSeq2500 machine with a TruSeq SBS sequencing kit version 1 analysed with Casava v1.8.2. Raw reads were filtered for duplicates using a custom script and trimmed for 5′ bias and 3′ quality using the FASTX-toolkit [64]. Transcriptome was assembled using SOAPdenovo-Trans v1.02 [65] with a k-mer of 49.

### Genome size estimation by flow cytometry

Genome size estimates were obtained through flow cytometry [66]. A total of 22 individuals were analysed, seven females and six males of *P. spumarius,* and nine females of *P. maghresignus*, a closely related species of the same genus. A suspension of nuclei from both the *Philaenus* sample and a reference standard (*Solanum lycopersicum*, S.l., ‘Stupické’ with 2C = 1.96 pg; [67]) were prepared by chopping the thorax and the head of the insect together with 0.5 cm^2^ of *S. lycopersicum* fresh leaf with a razor blade in a Petri dish containing 1 mL of WPB (0.2 M Tris. HCl, 4 mM MgCl_2_.6H_2_O, 1% Triton X-100, 2 mM EDTA Na_2_.2H_2_O, 86 mM NaCl, 10 mM metabisulfite, 1% PVP-10, pH adjusted to 7.5 and stored at 4°C; [68]). The nuclear suspension was filtered through a 30 μm nylon filter and 50 μg mL^−1^ of propidium iodide (PI, Fluka, Buchs, Switzerland) and 50 μg mL^−1^ of RNAse (Fluka, Buchs, Switzerland) were added to stained DNA and avoid staining of double stranded RNA, respectively. After 5 minutes of incubation, the nuclear suspension was analysed in a Partec CyFlow Space flow cytometer (532 nm green solid-state laser, operating at 30 mW; Partec GmbH., Görlitz, Germany). Data was acquired using the Partec FloMax software v 2.4d (Partec GmbH, Münster, Germany) in the form of four graphics: histogram of fluorescence pulse integral in linear scale (FL); forward light scatter (FS) vs. side light scatter (SS), both in logarithmic (log) scale; FL vs. time; and FL vs. SS in log scale. To remove debris, the FL histogram was gated using a polygonal region defined in the FL vs. SS histogram. At least 1,300 nuclei were analysed per *Philaenus*’ G_1_ peak [69]. Only CV values of 2C peak of *Philaenus* below 5% were accepted [70]. The homoploid genome size (2C in pg; [71]) was assessed through the formula: sample nuclear DNA content (pg) = (sample G_1_ peak mean/*S. lycopersicum* G_1_ peak mean) * genome size of *S. lycopersicum*. The obtained values were expressed in picograms (pg) and in giga base pairs (Gb), using the formula by [72] (1 pg = 0.978 Gb).

Differences in genome size between males and females were evaluated using a one-way analysis of molecular variance (ANOVA), followed by a Tukey test for multiple comparisons at *P <* 0.05. Statistical analyses were performed using SigmaPlot for Windows v. 12.5 (Systat Software).

### Characterisation of RAD loci

A consensus sequence, with IUPAC ambiguity codes for variable sites, was generated for each RAD locus across individuals using the python script loci_consensus.py [73].

Homology to non-coding and coding regions was investigated for the inferred loci by locally querying consensus sequences against Arthropoda sequences available in the NCBI nucleotide database (RefSeq release 73, last modified 2 November 2015 and GenBank release 211, last modified 14 December 2015), using BLASTN 2.2.28+ [74]. A protein blast (RefSeq release 73, last modified 2 November 2015 and GenBank release 211, last modified 14 December 2015), using BLASTX 2.2.28+ [75], was also performed. An E-value threshold of 1e-5 was used.

RAD loci were also queried using BLASTN against the drafts of the *P. spumarius* genome and transcriptome assembled in this study. In this case, an E-value threshold of 1e-15 was chosen as the cutoff for restricting the alignments to the most significant ones. The top five contigs and/or scaffolds were subsequently investigated by querying them using BLASTN against Arthropoda sequences available in nucleotide and protein databases of NCBI.

## Results

### RAD sequencing and SNPs data matrix

The sequencing set produced a total of 341 million reads. After filtering reads based on quality scores, 269 million reads were retained, corresponding to an average of 7.4 million reads per individual. Before filtering, individuals yielded 335,767 to 12,711,816 sequenced reads of 90 bp each (Additional file 1: Figure S1).

The average number of reads per locus per individual used to estimate a consensus sequence was 51.0 (Additional file 1: Figure S2). For the clustering results, a total of 133,127 loci, consisting of 12,144,351 aligned nucleotides, inferred with a minimum of nine individuals (25%) per locus, and a total of 470,470 SNPs with a mean percentage of missing data per individual of 63.92%, were produced. Aligned loci, including gaps inserted in the course of the alignment, ranged from 90 to 109 bp in length (mean = 91 bp). When filtering by percentage of missing data, three individuals (TYP_5, TYP_13 and TRI_13; Additional file 1: Figure S1, S2 and S3) had more than 90% missing data and were excluded. After filtering, a set of 928 loci, 85,056 bases and 2,195 SNPs was retained. However, only 1,837 SNPs on 928 loci were considered for the analyses after those in the same locus sequence with a complete LD (*r* ^2^ = 1) were randomly excluded.

### Single-SNP associations with colour phenotypes

**Fig. 1:**
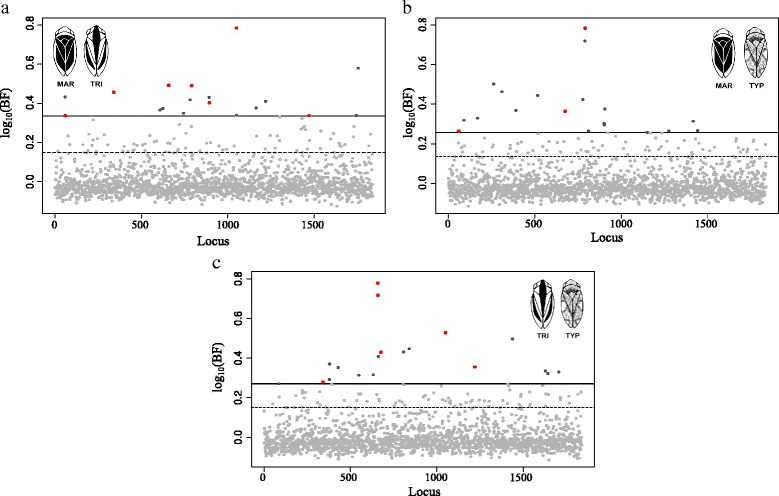
Bayes factor for each SNP in each pairwise comparison in single-SNP association tests. **a** MAR vs. TRI; **b** MAR vs. TYP; and **c** TRI vs. TYP. The horizontal dash lines correspond to the Bayes factor 95% empirical quantile threshold and the straight lines to the 99% empirical quantile. Light grey dots: SNPs with a BF < 99% empirical quantile; Dark grey dots: SNPs with a BF > 99% empirical quantile; Red dots: SNPs with a BF > 99% empirical quantile and shared among comparisons

**Fig. 2:**
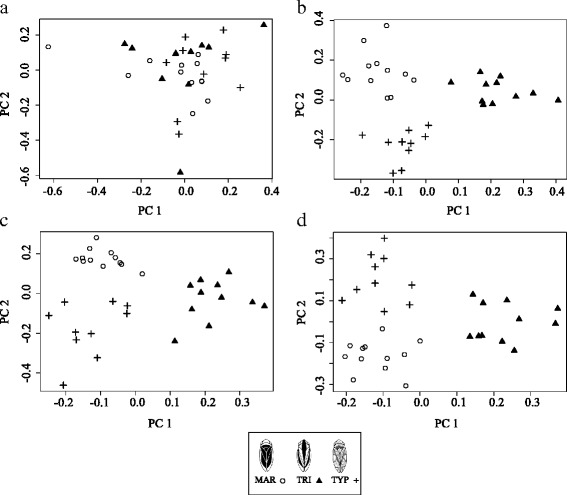
Genetic variation of the 33 individuals summarised on principal component axis 1 (PC1) and 2 (PC2) from a Principal Component Analysis using SNPs identified through Bayesian regression analyses. **a** All 1,837 SNPs; **b** 50 SNPs BF_0.99_ identified in Single-SNP association tests; **c** 50 SNPs PIP_0.99_ identified in Multi-SNP Association tests; and **d** 40 SNPs shared between both association analyses

The dataset was tested for allele frequency differences between pairs of dorsal colour phenotypes – MAR vs. TYP, TRI vs. TYP and MAR vs. TRI – using the Fisher’s exact test and a Bayesian regression approach. Single-marker association analyses performed using the frequentist method found 205 SNPs with *p*-value < 0.05, corresponding to 11.16% of the analysed SNPs, but these were not significant after FDR correction (Additional file 2: Table S1). Single-SNP analyses using the Bayesian regression approach identified a total of 230 BF_0.95_ SNPs (>95% quantile Bayes Factor) associated with dorsal colour phenotypes, corresponding to 12.52% of the analysed markers. When a more strict, 99% quantile, threshold was applied 50 BF_0.99_ SNPs (2.7%) showed the strongest associations to colour morphs, including eight shared among colour morph comparisons (Fig. 1) (Table 1) The number of BF_0.95_ SNPs and BF_0.99_ SNPs for each pairwise comparison were: 92 and 19, respectively, for MAR-TYP; 92 and 20, respectively, for TRI-TYP; 101 and 19, respectively, for MAR-TRI. Estimates of the phenotypic effects associated with BF_0.99_ SNPs for each comparison were moderate with 0.10 < | β | < 0.15 but much higher than the overall average for each pairwise analysis (| β | = 0.0001, MAR-TRI; | β | = 0.0037, MAR-TYP; | β | = 0.0028, TRI-TYP) (Table 1). Allele frequencies for the 50 SNPs involved in the differentiation of these colour morphs varied across the three colour phenotypes (Table 1). For the 50 BF_0.99_ SNPs, *F*_*ST*_ estimates between pairs of colour morphs were highly significant (*p*-value < 0.0001) (Additional file 2: Table S2), with the highest genetic differentiation between TRI and MAR (*F*_*ST*_ = 0.2145), intermediate between TRI and TYP (*F*_*ST*_ *=* 0.2125) and the lowest between MAR and TYP (*F*_*ST*_ = 0.1787) (Additional file 2: Table S3). Principal Component Analysis using the associated BF_0.99_ SNPs showed a clear distinction among the three morphs when compared with the PCA using all 1,837 SNPs (Fig. 2a). Principal component 1 explained 13% of the total variation and indicated a differentiation between TRI and the other two colour morphs while PC2 explained 10% of the differences, separating TYP from MAR (Fig. 2b).

**Table 1:** SNPs associated with dorsal colour morphs for each pairwise comparison and obtained through Single-SNP association tests using Bayesian regression approach

SNP_ID	Minor Allele	Major Allele	BF_0.99_	β	maf_TRI	maf_MAR	maf_TYP
MAR-TRI							
3950:1	G	A	0.432	−0.1198	0.1667	0.7727	0.3750
3950:10	T	A	0.337	−0.1099	0.1667	0.7273	0.3125
22795:88	G	A	0.457	−0.1246	0.0500	0.5000	0.4286
40633:18	A	G	0.365	0.1159	0.4500	0.0000	0.0714
41239:75	G	T	0.373	0.1170	0.3333	0.0000	0.1000
43069:10	C	G	0.492	−0.1305	0.0000	0.4167	0.5714
43069:34	A	T	0.492	−0.1305	0.0000	0.4167	0.5000
50515:83	A	T	0.349	0.1165	0.2857	0.0000	0.0000
54226:66	A	T	0.418	−0.1245	0.0000	0.3500	0.0000
55187:79	G	T	0.490	0.1303	0.3889	0.0000	0.5000
63439:28	C	A	0.403	−0.1203	0.1818	0.6000	0.3889
63439:8	A	C	0.430	−0.1224	0.1364	0.5500	0.3500
75897:50	A	G	0.339	0.1158	0.2500	0.0000	0.0000
75897:7	C	T	0.785	0.1581	0.5833	0.0000	0.0000
83460:19	C	T	0.376	−0.1173	0.0000	0.3889	0.0000
87932:85	C	T	0.410	−0.1238	0.0000	0.5000	0.5556
106126:52	C	T	0.337	0.1161	0.2500	0.0000	0.0000
124817:20	A	G	0.338	0.1159	0.3500	0.0000	0.0625
126355:29	T	C	0.579	0.1369	0.5500	0.0455	0.1667
Mean BF0.99 SNPs				0.1235			
Mean all SNPs				0.0001			
MAR-TYP							
3950:10	T	A	0.264	−0.1010	0.1667	0.7273	0.3125
7095:50	C	T	0.319	0.1123	0.0556	0.0417	0.2000
11381:9	A	G	0.329	0.1131	0.1667	0.0000	0.2857
16628:65	C	A	0.502	−0.1287	0.0000	0.5000	0.0000
20734:39	T	C	0.463	0.1275	0.4286	0.3000	0.7778
24668:63	C	G	0.369	0.1173	0.3333	0.3182	0.7500
35205:6	G	C	0.444	0.1255	0.0625	0.2273	0.0000
45009:87	T	G	0.365	0.1096	0.1500	0.1818	0.6500
54049:70	G	A	0.424	0.1231	0.5000	0.2727	0.7500
55187:46	A	G	0.719	0.1555	0.2778	0.0000	0.5000
55187:79	G	T	0.784	0.1603	0.3889	0.0000	0.5000
56842:83	A	G	0.265	0.1053	0.2000	0.0455	0.2500
64204:16	T	G	0.303	−0.1080	0.1250	0.5000	0.1111
64204:46	G	T	0.296	−0.1072	0.2778	0.5000	0.1111
64258:61	G	A	0.376	0.1207	0.1500	0.0455	0.4286
82682:38	T	G	0.258	0.1019	0.4000	0.1111	0.5833
92187:65	A	C	0.265	−0.1028	0.5000	0.6111	0.1875
102702:13	T	A	0.314	0.1120	0.0000	0.0000	0.2143
104139:11	T	A	0.267	−0.1040	0.3500	0.7000	0.3333
Mean BF0.99 SNPs				0.1177			
Mean all SNPs				0.0037			
TRI-TYP							
6535:26	T	C	0.270	−0.1035	0.4444	0.2222	0.0000
6535:35	G	A	0.270	0.1023	0.1667	0.5556	0.6250
22795:88	G	A	0.279	0.1075	0.0500	0.5000	0.4286
24031:66	C	T	0.292	0.1099	0.0000	0.1111	0.2000
24031:81	T	G	0.370	0.1202	0.0000	0.0556	0.2000
27816:86	G	A	0.352	−0.1155	0.6364	0.4375	0.1875
37095:26	T	G	0.313	−0.1110	0.4444	0.1000	0.0556
41742:86	C	G	0.316	0.113	0.0000	0.4167	0.5714
43069:10	C	G	0.778	0.1577	0.0000	0.4167	0.5714
43069:34	A	T	0.717	0.1551	0.0000	0.4167	0.5000
43143:5	T	C	0.408	−0.1195	0.7222	0.4000	0.2500
45009:87	T	G	0.429	0.1180	0.1500	0.1818	0.6500
56752:20	G	A	0.431	−0.1223	0.6875	0.4444	0.1250
59359:24	G	A	0.447	0.1285	0.1000	0.2000	0.6000
75897:7	C	T	0.528	−0.1342	0.5833	0.0000	0.0000
87932:85	C	T	0.355	0.1135	0.0000	0.5000	0.5556
103746:74	T	A	0.496	0.1342	0.0909	0.4000	0.5833
118051:49	G	C	0.335	−0.1170	0.1000	0.1500	0.0000
118835:54	C	A	0.322	−0.1122	0.0455	0.3636	0.0000
123202:88	T	A	0.330	0.1130	0.0909	0.1000	0.4286
Mean BF0.99 SNPs				0.1204			
Mean all SNPs				0.0028			

Bayes factor values above 0.99 quantile (BF_0.99_); Effect size of an individual SNP on the phenotype (β); Minor allele frequency for each locus and morph (maf); Mean effect size of BF_0.99_ SNPs (Mean BF_0.99_ SNPs); Mean effect size of all 1,837 SNPs. SNPs common to comparisons are underlined

### Multi-SNP Associations with colour phenotypes

**Fig. 3:**
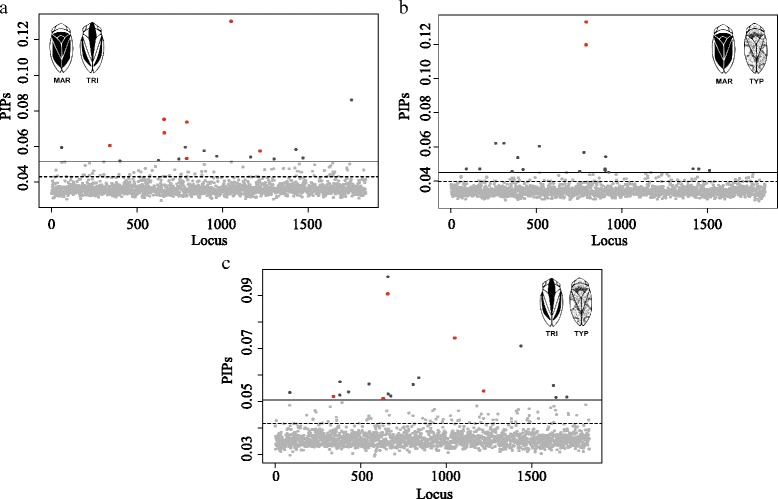
Posterior inclusion probabilities (PIPs) for each SNP in each pairwise comparison in multi-SNP association tests. **a** MAR vs. TRI; **b** MAR vs. TYP; and **c** TRI vs. TYP. The horizontal dash lines correspond to the PIP 95% empirical quantile threshold and the straight lines to the 99% empirical quantile. Light grey dots: SNPs with a PIP < 99% empirical quantile; Dark grey dots: SNPs with a PIP > 99% empirical quantile; Red dots: SNPs with a PIP > 99% empirical quantile and shared among comparisons

The 1,837 SNPs dataset explained between 60 and 65% of the variance in dorsal colour phenotypes across all pairwise analyses of colour morphs. The highest proportions of variation explained by the investigated SNPs were detected in comparisons involving the TRI phenotype (Table 2). The highest proportion was observed in TRI-TYP analysis (*PVE* = 0.6515) while the lowest proportion was found in MAR-TYP analysis (*PVE* = 0.6018) (Table 2). Estimates of the mean number of SNPs (nSNPs) underlying dorsal colour variation ranged from 63 to 67 (Table 2). However, 95% credible intervals for these parameters estimates were typically large. The average effect of associated SNPs was high and similar among analyses but once again higher in comparisons involving TRI (σSNP = 1.1200, MAR-TRI; σSNP = 0.9776, TRI-TYP; σSNP = 0.9495, MAR-TYP) (Table 2). When considering models with the highest BFs (log_10_(BF) > 10) only, the mean number of SNPs included in the model (nSNPs_BF) for each comparison decreased up to values between nine and 12 while the mean effect size of the SNPs (σSNP_BF) increased ranging between 2.4 and 4.1 (Table 2). The posterior inclusion probabilities (PIPs) for the analysed SNPs were quite similar among all pairwise analyses but slightly higher in comparisons involving TRI (PIP = 0.0366, MAR-TRI; PIP = 0.0362, TRI-TYP and PIP = 0.0345, MAR-TYP) (Fig. 3) (Table 2). A subset of 19 SNPs with the highest inclusion probabilities (PIP_0.99_ SNPs) were identified for each analysis and investigated (Table 3). This number was within the 95% credible intervals for the number of SNPs found to be associated with dorsal colour variation by the models with the highest BF (Additional file 1: Figure S4) (Table 3). Estimates of the strength of association between genotypic variation at individual SNPs and phenotypic variation (| β |) varied among the analyses and all were greater than 0.5. We obtained SNPs with larger effect sizes for MAR-TRI analysis than for all other analyses. Six PIP_0.99_ SNPs were shared between two pairwise analyses (Table 3). In total, 50 different SNPs revealed a multi-association with colour morphs and, from those, 40 were also significant in the single-SNP analyses shown previously. For the 50 PIP_0.99_ SNPs, population differentiation tests were also highly significant (*p*-value < 0.000) (Additional file 2: Table S2). Similarly, the highest genetic differentiation was observed between TRI and TYP (*F*_*ST*_ = 0.2159), intermediate between TRI and MAR (*F*_*ST*_ = 0.1907) and the lowest genetic differences were observed between MAR and TYP (*F*_*ST*_ = 0.1650) (Additional file 2: Table S3). Principal Component Analysis for all 50 PIP_0.99_ SNPs of multi-association tests (Fig. 2c) and for the 40 intersected SNPs (Fig. 2d) showed the expected differentiation among dorsal colour morphs. Principal Component 1 explained 13 to 14% of the variance, differentiating TRI from the other morphs while PC2 explained 11% of the differences and revealed a differentiation between TYP and MAR.

**Table 2:** Parameter estimates from Bayesian variable selection regression for each pairwise analysis

Analyses	PVE	σSNP	σSNP_BF	nSNP	nSNP_BF	PIP SNP
MAR-TRI	0.6429 (0.031–0.998)	1.1200 (0.0570–5.559)	3.4300 (0.8475–11.8320)	67 (1–268)	12 (2–31)	0.366 (0.0320–0.0465)
MAR-TYP	0.6018 (0.027–0.995)	0.949 (0.0520–4.0220)	2.4070 (0.8531–7.2788)	63 (1–264)	9 (2–26)	0.0345 (0.0303–0.0418)
TRI-TYP	0.6515 (0.035–0.996)	0.9776 (0.0570–4.4040)	4.1420 (0.6660–8.7020)	66 (1–263)	10 (2–25)	0.0361 (0.0320–0.0448)

Proportion of variance explained (PVE); mean phenotypic effect associated with a SNP in the regression model including all models (σSNP) and models with a log_10_(BF) > 10 (σSNP_BF); mean number of SNPs in the model considering all models (nSNP) and models with a log_10_(BF) > 10 (nSNP_BF) and; mean posterior inclusion probability associated to SNPs in the model (PIP). 95% empirical quantiles are reported in parenthesis

**Table 3:** SNPs PIP_0.99_ associated with dorsal colour morphs obtained through Multi–SNP association tests using Bayesian regression approach

MARTRI							
SNP_ID	PIP	β	Minor Allele	Major Allele	MAF TRI	MAF MAR	MAF TYP
41239:75	0.05228	0.73688	G	T	0.3333	0.0000	0.1000
50515:83	0.05294	0.70499	A	T	0.2857	0.0000	0.0000
55187:46	0.05326	0.67185	A	G	0.2778	0.0000	0.5000
55187:79	0.07371	1.18427	G	A	0.3887	0.0000	0.5000
69098:53	0.0545	4.0856	C	A	0.1818	0.0000	0.2778
75897:7	0.13035	0.89186	C	T	0.5833	0.0000	0.0000
94147:30	0.05298	0.7147	G	A	0.6818	0.3182	0.3889
106126:52	0.05356	1.10651	C	T	0.2500	0.0000	0.0000
126355:29	0.08618	3.89385	T	C	0.5500	0.0455	0.1667
3950:1	0.05946	−0.86693	G	A	0.1667	0.7727	0.3750
22795:88	0.06048	−0.98089	G	A	0.0500	0.5000	0.4286
25027:11	0.05188	−1.28702	T	A	0.0000	0.1667	0.1667
3950:1	0.05946	−0.86693	G	A	0.1667	0.7727	0.3750
22795:88	0.06048	−0.98089	G	A	0.0500	0.5000	0.4286
25027:11	0.05188	−1.28702	T	A	0.0000	0.1667	0.1667
43069:10	0.07528	−2.3343	C	G	0.0000	0.4167	0.5714
43069:34	0.06763	−1.50904	A	T	0.0000	0.4167	0.5000
54226:66	0.05956	−1.00561	A	T	0.0000	0.3500	0.0000
63439:8	0.05754	−0.73234	A	C	0.1364	0.5500	0.3500
83460:19	0.05406	−0.72601	C	T	0.0000	0.3889	0.0000
87932:85	0.05747	−0.72742	C	T	0.0000	0.5000	0.5556
103246:16	0.05827	−1.14876	T	C	0.0000	0.3571	0.1000
TRITYP							
SNP_ID	PIP	β	Minor Allele	Major Allele	MAF TRI	MAF MAR	MAF TYP
6535:35	0.0534	0.92979	G	A	0.1667	0.5556	0.6250
22795:88	0.0519	0.71932	G	A	0.0500	0.5000	0.4286
24031:66	0.05241	1.12531	C	T	0.0000	0.1111	0.2000
24031:81	0.05745	0.84617	T	G	0.0000	0.0556	0.2000
41742:86	0.05111	0.74532	C	G	0.0000	0.4167	0.5714
43069:10	0.09056	1.35007	A	T	0.0000	0.4167	0.5000
43069:34	0.09699	1.79618	A	T	0.0000	0.4167	0.5000
45009:87	0.05206	0.64932	T	G	0.1500	0.1818	0.6500
59359:24	0.05896	1.0716	G	A	0.1000	0.2000	0.6000
87932:85	0.05394	0.98257	C	T	0.0000	0.5000	0.5556
103746:74	0.07094	1.25641	T	A	0.0909	0.4000	0.5833
123202:88	0.05166	0.6811	T	A	0.0909	0.1000	0.4286
27816:86	0.05363	−0.79657	G	A	0.6364	0.4375	0.1875
37095:26	0.05663	−0.84607	T	G	0.4444	0.1000	0.0556
43143:5	0.05286	−0.67184	T	C	0.7222	0.4000	0.2500
56752:20	0.05638	−0.73623	G	A	0.6875	0.4444	0.1250
75897:7	0.07396	−1.31379	C	T	0.5833	0.0000	0.0000
118051:49	0.05605	−0.82887	G	C	0.1000	0.1500	0.0000
118835:54	0.05156	−0.68197	C	A	0.0455	0.3636	0.0000
MARTYP							
SNP_ID	PIP	β	Minor Allele	Major Allele	MAF TRI	MAF MAR	MAF TYP
7095:50	0.04707	0.65574	C	T	0.0556	0.0417	0.2000
11381:9	0.04714	0.61586	A	G	0.1667	0.0000	0.2857
20734:39	0.0621	0.06957	T	C	0.4286	0.3000	0.7778
23155:83	0.04559	0.58512	T	G	0.1111	0.1000	0.5000
24668:63	0.05327	0.84822	C	G	0.3333	0.3182	0.7500
27059:59	0.04681	1.00875	T	C	0.2222	0.2083	0.5000
35205:6	0.06037	0.89098	G	C	0.0625	0.2273	0.0000
54049:70	0.0568	0.76067	G	A	0.5000	0.2727	0.7500
55187:46	0.11972	1.88694	A	G	0.2778	0.0000	0.5000
55187:79	0.13314	0.66567	G	T	0.3889	0.0000	0.5000
64258:61	0.05429	0.96162	G	A	0.1500	0.0455	0.4286
102702:13	0.04718	0.03275	T	A	0.0000	0.0000	0.2143
104623:88	0.04711	0.70632	G	A	0.1875	0.0000	0.1429
108304:78	0.04626	0.48985	C	A	0.3571	0.2971	0.6250
16628:65	0.06209	−0.89022	C	A	0.0000	0.5000	0.0000
51349:15	0.04563	−0.59847	T	C	0.1667	0.2500	0.0000
64204:16	0.04703	−0.60862	T	G	0.1250	0.5000	0.1111
64204:46	0.04626	−0.59146	G	T	0.2778	0.5000	0.1111
66105:38	0.04521	−0.53359	A	C	0.3000	0.2143	0.0500

Posterior inclusion probability associated to SNP (PIP); Effect size of an individual SNP on the phenotype (β) and minor allele frequency for each locus and morph (maf). SNPs common to comparisons are underlined

### Linkage patterns

The associated loci detected here had on average low levels of linkage disequilibrium for both analyses including all samples or analyses on each colour phenotype separately (Additional file 1: Figure S5). However, strong allelic correlations (*r* ^2^ > 0.7) were found for five pairs of SNPs within MAR and for two pairs in TYP phenotypes (Additional file 2: Table S4). Only two pairs, in MAR, consisted of SNPs present in the same RAD locus.

### Genome size estimation

*Philaenus spumarius* and *P. maghresignus* estimates of genome size were 5.27 ± 0.25 pg (5.15 Gb) and 8.90 ± 0.20 pg (8.90 Gb), respectively. In *P. spumarius*, males and females differed significantly in genome size (*F*_1,11_ = 14.292, *p*-value = 0.0030), with males presenting on average a lower genome size (5.07 ± 0.20 pg; 4.96 Gb) than females (5.44 ± 0.15 pg; 5.33 Gb) (Additional file 2: Table S5). Overall, the quality of the analyses was excellent, with a mean CV value of 2.97% being obtained for the sample’s G_1_ peak.

### *De novo* sequencing and assembly of meadow spittlebug genome and transcriptome

The genome sequencing set produced a total of 366 million reads. After filtering reads based on quality, 353 million reads (96.46%) were retained (Additional file 2: Table S6). SOAPdenovo2 produced 6,843,324 *contigs* and 4,010,521 scaffolds. The N50 was 686 bp and the percentage of gaps was 20.47%. In total, 1,218,749,078 bp were assembled which based on the total estimated genome size of 5.3 Gb, corresponds to approximately 24% of the *P. spumarius* genome.

For the transcriptome, the total number of 150 nt reads for each paired-end of the library was 17 million resulting in 5110.8 Mb of sequence (Additional file 2: Table S6). After quality filtering, 14 million (86.81%) read pairs were used in the assembly (Additional file 2: Table S6). The transcriptome assembly produced 173,691 contigs and 31,050 scaffolds. In this case, the observed N50 obtained was 803 bp and the percentage of gaps 0.39%. A total of 81,442,967 bp were assembled. Assembly statistics for the genome and transcriptome are summarised in Additional file 2: Table S6.

### Characterisation of RAD loci

No significant hits were found when querying the 928 RAD loci against Arthropoda sequences of NCBI nt database and only 15 hits (E-value < 1e-05) were found against Arthropoda sequences of NCBI nr database (Additional file 2: Table S7). However, this was not unexpected considering RAD loci sequences are less than 100 bp and the most closely related insect species with an available genome is the pea aphid *Acyrthosiphon pisum*, which belongs to a separate hemipteran infraorder.

A total of 392 RAD loci (42.24%) aligned to the draft of *P. spumarius* genome (E-value threshold of 1e-15), 18 of which were associated with colour morphs (34.62% of the colour-associated loci sequences) (Additional file 2: Table S8). On the other hand, 134 loci, corresponding to 14.44% of the total loci, aligned to *P. spumarius* transcriptome assembly. Five of those were colour-associated (9.62% of the colour-associated loci) (Additional file 2: Table S8). From the 18 colour-associated loci that aligned with the genome, four (22.22%) also aligned with the transcriptome. The proportion of colour-associated loci that aligned either with the genome or with the transcriptome was not significantly different from the proportions of the other loci (Fisher’s exact test *p*-value = 0.8096). Some RAD loci had more than one contig/scaffold hit (Additional file 1: Figure S6).

Transcriptome and genome scaffolds/contigs with RAD loci alignments, ranging from 100 to 12,325 bp (gaps included), were queried against Arthropoda nt and nr databases using BLASTN and BLASTX. Out of 210 transcriptome sequences, 22 (E-value < 1e-05) had homology with the nucleotide database (Additional file 2: Table S9) and 98 with the protein database (Additional file 2: Table S10). The majority of those sequences hits have E-values < 1e-12 in nucleotide (86.36%) and in protein (69.38%) blasts. On the other hand, one genome scaffold, out of 484 with RAD loci hits, matched with the nucleotide sequences (E-value < 1e-25) (Additional file 2: Table S11) and 90 with the protein database (Additional file 2: Table S12). The majority of those protein hits have E-values < 1e-12 (55.55%). Of the transcriptome and genome sequences with protein hits, five and three included associated loci, respectively (Additional file 2: Table S13). Four of these genome and transcriptome sequences matched with two known proteins, the other four with uncharacterised ones. One of the identified proteins, to which the colour-associated locus 16628 aligned (genome scaffold 1372429 and transcriptome scaffolds 17697 and 17698), was a lysosomal-trafficking regulator, known to be involved in the trafficking of materials into lysosomes. Furthermore, a mutation of this protein in humans is associated with a pigmentation disorder [76]. The other identified protein, to which the colour-associated locus 22795 aligned (transcriptome scaffold 29739), was the nucleolar and coiled-body phosphoprotein 1. This locus is one of the eight shared among colour morph comparisons.

## Discussion

In this study, we aimed to identify candidate genomic regions associated with colour polymorphism in the meadow spittlebug *P. spumarius*, an insect species with a very large genome (5.3 Gb), as estimated here by flow cytometry. This large size is among the largest genomes reported in insects [77], making genomic analysis in this species particularly challenging. By using restriction site-associated DNA (RAD) sequencing in individuals of three dorsal colour phenotypes (*typicus*, *marginellus,* and *trilineatus*), we were able to detect association with colour in 3% of the analysed SNPs (60 out of 1,837). These phenotypes did not reveal significant genome-wide differences but when considering only the associated SNPs, the three colour morphs were differentiated and the *trilineatus* phenotype showed the highest genetic differentiation. Interestingly, greater differences involving life-history traits such as longevity, number of eggs, and number of oviposition events are also known to occur in *trilineatus* [33]. It may be that the genetic differences detected in this morph also reflect some part of the genetic basis of these life-history differences among colour morphs. Therefore, we may not only be on the track to finding a colour gene but also perhaps an extensive region, or several regions of the genome, that links colour variation and other life-history or physiological traits, as previously suggested [38]. Our finding of several colour-associated SNPs, some of them mapped to different genome and transcriptome scaffolds, suggests a complex genetic architecture involving this colour polymorphism.

In the single-SNP association analyses, the 50 individual SNPs found to be associated at 99% quantile (BF_0.99_ SNPs) showed moderate phenotypic effects (0.10 < | β | < 0.15). In the multi-SNP association analyses, 50 SNPs with posterior inclusion probabilities at quantile 99% (PIP_0.99_ SNPs) showed large effects for pairs of colour phenotypes (σSNP > 0.9 and individual PIP_0.99_ SNPs | β | > 0.5). From these, 40 were common to the SNPs identified in single SNP analyses (BF_0.99_ SNPs), increasing the confidence for the detected associations. Although inferences about the genetic architecture are only tentative in this study, due to the relatively small proportion of the genome covered, our results suggest that differences among the three dorsal colour phenotypes are associated with several loci with large effects. However, it is still not entirely clear if these constitute the major locus determining dorsal colour pattern, revealed by Mendelian crosses in *P. spumarius* [31, 36]. Large effect loci controlling colour pattern have been reported for *Heliconius* species [78, 79], land snail *Cepaea nemoralis* [42], and more recently in *Timema cristinae* stick insects [80]. Several other examples [81–83] have shown that adaptive traits are affected by loci with large phenotypic effects and that this genetic architecture may be more common than initially thought. The majority of the colour-associated loci that we detected here did not show significant allelic correlations, being likely in independent genomic regions. However, a few loci were strongly correlated, indicating either physical linkage, random drift of rare alleles, or occurrence of recent mutations. The existence of tightly linked loci (a supergene) that can be maintained due to chromosomal rearrangements or selection of co-adapted loci with epistatic effects is also possible. In the mimetic butterfly *Heliconius melpomene*, a cluster of three tightly linked loci (HmN, HmYb and HmSb), lying just a few centimorgans apart, as well as other unlinked loci have been shown to control distinct wing colour pattern elements in this species [84]. In a closely related species *Heliconius numata,* polymorphic colour variation is controlled by a single locus *P,* forming a supergene, resulting from chromosomal rearrangements [85]. A single gene, *doublesex*, with closely linked mutations, also controls supergene mimicry in *Papilio polytes* [86].

Various genes and pathways have been reported to be involved in insect coloration and pigmentation. These pathways comprise genes regulating the distribution of pigments in space and time, as well as genes that are involved in the synthesis of pigments [87]. Several colour genes have been described, mostly in *Drosophila* spp. (see [87] for a review) and are known to be involved in colour variation in the silk worm *Bombyx mori* [88] and *Papilio* spp. [86, 89, 90] as well. Novel and unexpected genes were found to be responsible for wing colour patterning in *Heliconius* species. Red wing elements are associated with expression of the transcription factor *optix* [91], which in turn is regulated by two distinct *cis*-regulatory loci [92]. Another gene, *cortex*, a member of a conserved cell cycle regulator family, appears to have adopted a novel function controlling colour pattern in *Heliconius* and probably across the Lepidoptera [93]. Regulatory regions are also known to control colour pattern in *Drosophila* flies [94, 95]. However, none of the colour-associated loci that we found in our study matched these candidate genes and/or genomic regions of other insects. Approximately 10% of the loci with colour associations aligned with the *P. spumarius* transcriptome indicating that those loci are in coding regions that are expressed in adult stage. A similar proportion of alignment to the transcriptome was found between associated loci and all loci (Fisher’s exact test *p*-value = 0.8096), suggesting that there is no enrichment/depletion of coding regions in the associated loci in relation to the total number of loci. Around 35% of the colour-associated loci aligned with the genome and 22% also aligned with the transcriptome. If we assume a good representation of the total transcriptome, this result point to the majority of the associated loci being in non-coding regions. Considering that our assembled genome represents only 24% of the total genome size, the low percentage of hits in the genome was expected. Also, the low number of nucleotide and protein matches of genome and transcriptome sequences is certainly due to the degree of similarity of *P. spumarius* to other available Arthropoda sequences being too low to allow significant matches. Increasing the genomic resources for this or related species will allow exploring the candidate loci here described and provide insight into some of the key questions that remain to be answered. What are the specific genes contributing to this balanced colour polymorphism? What mutations cause allelic differences in these genes and how do they contribute to the different colour phenotypes? Are there epistatic or additive effects among the alleles responsible for the polymorphism? Does this constitute a supergene? Are coding or regulatory mutations involved? In the future, it would also be interesting to investigate the evolutionary history of the colour polymorphism within *Philaenus* since identical variation in dorsal colour/pattern can be observed in the other species of the genus, suggesting an ancestral polymorphism maintained through the speciation process.

## Conclusions

This work was a first approach to investigate the genetic architecture of *P. spumarius* dorsal colour polymorphism, by studying single and multi-SNP association with three of the colour phenotypes. We detected several loci with large effects occurring in multiple genomic regions. *Trilineatus* was found to be the most differentiated colour phenotype for these loci, and since it is also the most differentiated for several life-history and physiological traits, we suggest that genetic bases of colour and of these traits are linked. The development of genomic and transcriptomic resources in this work was a first step toward characterizing these loci and will be very useful for further research on the genetic basis of dorsal colour pattern variation in *P. spumarius*.

## Additional files

Additional file 1: Figure S1 – S6. Histograms of the total number of raw reads, mean depth and proportion of missing data per individual and of the R^2^ values for each colour-associated SNP comparison; scatterplots of the number of SNPs in the model as a function of the Bayes factor for each pairwise comparison in multi-SNP association tests; and number of RAD loci hits with genome and transcriptome. (PDF 1411 kb)
Additional file 2: Table S1 – S13. Lists of colour-associated SNPs obtained for each pairwise comparison and association analyses; genic and genotypic differentiation tests; pairwise F_st_ estimates among dorsal colour phenotypes; SNP correlation value (r^2^) in linkage disequilibrium analyses; Genome size estimates; Assembly statistics for genome and transcriptome; and lists of blast results. (XLSX 159 kb)
Additional file 3: Data file with 1,837 SNPs. (VCF 287 kb)

## Abbreviations

AMOVA: Analysis of molecular variance
BF: Bayes Factor
BF_0.95_ SNPs: SNPs above 95% empirical quantile for BF
BF_0.99_ SNPs: SNPs above 99% empirical quantile for BF
BVSR: Bayesian variable selection regression
FDR: False Discovery Rate
FL: Histogram of fluorescence pulse integral in linear scale
FS: Forward light scatter
F_ST_: Fixation index
Gb: Giga base pairs
LD: Linkage disequilibrium
maf: Minor allele frequency
MAR: *marginellus*
nSNPs: Number of SNPs in the regression model
PCA: Principal Component Analysis
PCR: Polymerase Chain Reaction
pg: Picograms
PIP: Posterior inclusion probability
PIP_0.99_ SNPs: PIP greater than 99% empirical quantile
PVE: Proportion of variance explained
r^2^: Squared correlation coefficient
RAD: Restriction site-associated DNA sequencing
SNPs: Single Nucleotide Polymorphisms
SS: Side light scatter
TRI: *trilineatus*
TYP: *typicus*
β: Phenotypic effect
σSNP: Average phenotypic effect of a SNP that is in the model
