# Correction to: Assessing genotype-phenotype associations in three dorsal colour morphs in the meadow spittlebug *Philaenus spumarius* (L.) (Hemiptera: Aphrophoridae) using genomic and transcriptomic resources

**DOI:** 10.1186/s12863-020-00842-6

**Published:** 2020-03-24

**Authors:** Ana S. B. Rodrigues, Sara E. Silva, Francisco Pina-Martins, João Loureiro, Mariana Castro, Karim Gharbi, Kevin P. Johnson, Christopher H. Dietrich, Paulo A. V. Borges, José A. Quartau, Chris D. Jiggins, Octávio S. Paulo, Sofia G. Seabra

**Affiliations:** 1 Computational Biology and Population Genomics Group, cE3c – Centre for Ecology, Evolution and Environmental Changes, Departamento de Biologia Animal, Faculdade de Ciências da Universidade de Lisboa, Campo Grande, P-1749-016 Lisbon, Portugal; 2 Centro de Estudos do Ambiente e do Mar (CESAM), DBA/FCUL, Lisbon, Portugal; 3 Department of Life Sciences, Centre for Functional Ecology, University of Coimbra, Coimbra, Portugal; 4 Edinburgh Genomics, Ashworth Laboratories, King’s Buildings, The University of Edinburgh, Edinburgh, EH9 3JT UK; 5 Illinois Natural History Survey, Prairie Research Institute, University of Illinois, Champaign, IL USA; 6 Departamento de Ciências e Engenharia do Ambiente, Angra do Heroísmo, cE3c – Centre for Ecology, Evolution and Environmental Changes/Azorean Biodiversity Group and Universidade dos Açores, Açores, Portugal; 7 Department of Zoology, University of Cambridge, Downing Street, Cambridge, CB2 3EJ UK

**Correction to: BMC Genet (2016) 17:144**

**https://doi.org/10.1186/s12863-016-0455-5**

Following publication of the original article [1], it has been brought to the authors’ attention that in their paper (Rodrigues et al. 2016) they reported the genome size based on 2C values (diploid genome) when it is more common to present it as 1C value.

This has led to a misinterpretation of the percentage of the genome that was sequenced. However, none of the remaining analyses were affected.

Please find below the corrections to the text (organized per article PDF page number). The authors have also added a column with the 1C values in Additional file 2: Table S5.

Page 1, Abstract. Where it reads: “A partial genome assembly, representing 24% of the total size...”, it should read: “A partial genome assembly, representing 48% of the total size...”

Page 5 - Where it reads: “The homoploid genome size (2C in pg; [71]) was assessed through the formula”, it should read: “The holoploid genome size (2C in pg; [71]) was assessed through the formula”.

Page 5 - Where it reads: “The obtained values were expressed in picograms (pg) and in giga base pairs (Gb), using the formula by [72] (1 pg = 0.978 Gb)”, it should read: “The obtained values were expressed in picograms (pg) and in giga base pairs (Gb), using the formula by [72] (1 pg = 0.978 Gb). The monoploid genome size (1Cx) was obtained by dividing the holoploid genome size by the ploidy level, in this case, diploid, and was also expressed in picograms and giga base pairs.”

Page 9 - Where it reads: “*Philaenus spumarius* and P. maghresignus estimates of genome size were 5.27 ± 0.25 pg (5.15 Gb) and 8.90 ± 0.20 pg (8.90 Gb), respectively. In *P. spumarius*, males and females differed significantly in genome size (F1,11 = 14.292, p-value = 0.0030), with males presenting on average a lower genome size (5.07 ± 0.20 pg; 4.96 Gb) than females (5.44 ± 0.15 pg; 5.33 Gb) (Additional file 2: Table S5).”, it should read: “*Philaenus spumarius* and P. maghresignus estimates of monoploid genome size were 2.63 ± 0.13 pg (2.58 Gb) and 4.45 ± 0.10 pg (4.35 Gb), respectively. In *P. spumarius*, males and females differed significantly in monoploid genome size (F1,11 = 14.292, p-value = 0.0030), with males presenting on average a lower genome size (2.53 ± 0.10 pg; 2.47 Gb) than females (2.72 ± 0.08 pg; 2.66 Gb) (Additional file 2: Table S5).”

Page 10 - Where it reads: “In total, 1,218,749,078 bp were assembled which based on the total estimated genome size of 5.3 Gb, correspond to approximately 24% of the *P. spumarius* genome.”, it should read: “In total, 1,218,749,078 bp were assembled which based on the total estimated monoploid genome size of 2.58 Gb, correspond to approximately 48% of the *P. spumarius* genome.”

Page 12 - Where it reads: “... an insect species with a very large genome (5.3 Gb)...”, it should read: “... an insect species with a very large genome (2.58 Gb)...”

Moreover, the authors would like to inform the readers that the raw reads of the RAD libraries used for association analyses are available in NCBI under the accession PRJNA572593.

The authors apologize to the editor and readers for any inconvenience caused by this error.

Furthermore, they would like to thank Roberto Biello (John Innes Centre) and Saskia Hogenhout (John Innes Centre) for having brought this matter to their attention.

## Supplementary information

**Additional file 2: Table S1-S13.** Lists of colour-associated SNPs obtained for each pairwise comparison and association analyses; genic and genotypic differentiation tests; pairwise Fst estimates among dorsal colour phenotypes; SNP correlation value (r2) in linkage disequilibrium analyses; Genome size estimates; Assembly statistics for genome and transcriptome; and lists of blast results.
