# Supplementary material for: Assessing genotype-phenotype associations in three dorsal colour morphs in the meadow spittlebug Philaenus spumarius (L.) (Hemiptera: Aphrophoridae) using genomic and transcriptomic resources
Source: BMC Genet. 2016 Nov 15;17:144. doi: 10.1186/s12863-016-0455-5 (PMC5111214; doi:10.1186/s12863-016-0455-5)
Supplement: Additional file 1: Figure S1 – S6. — Histograms of the total number of raw reads, mean depth and proportion of missing data per individual and of the R2 values for each colour-associated SNP comparison; scatterplots of the number of SNPs in the model as a function of the Bayes factor for each pairwise comparison in multi-SNP association tests; and number of RAD loci hits with genome and transcriptome. (PDF 1411 kb) [file 12863_2016_455_MOESM1_ESM.pdf]

Additional File 1

Caption for entire file:

Histograms of the total number of raw reads, mean depth and proportion of missing data per individual and of the R^2 values for each colour-associated SNP comparison; scatterplots of the number of SNPs in the model as a function of the Bayes factor for each pairwise comparison in multi-SNP association tests; and number of hits with genome and transcriptome.

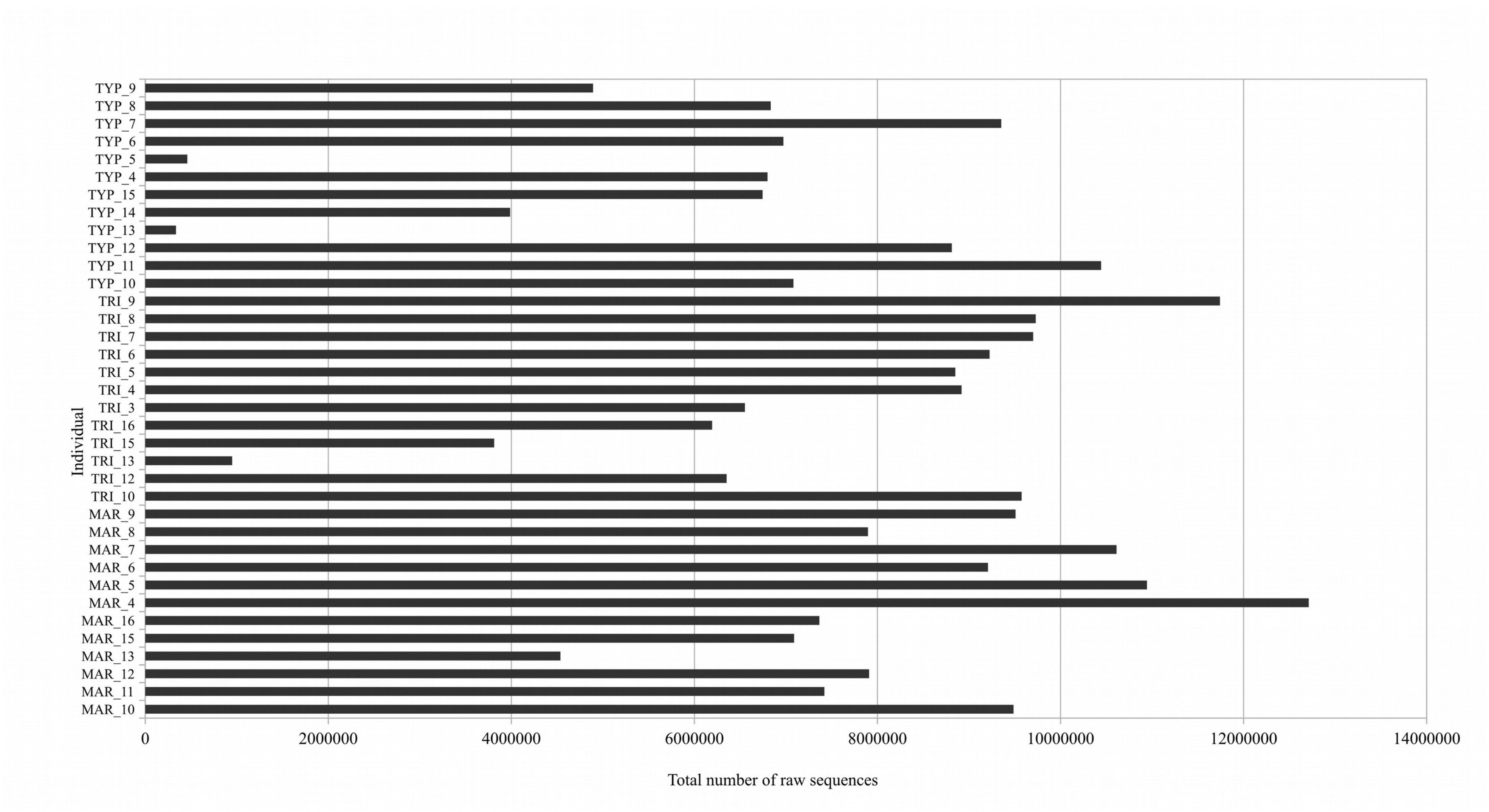

Figure S1 – Total number of raw reads per individual.

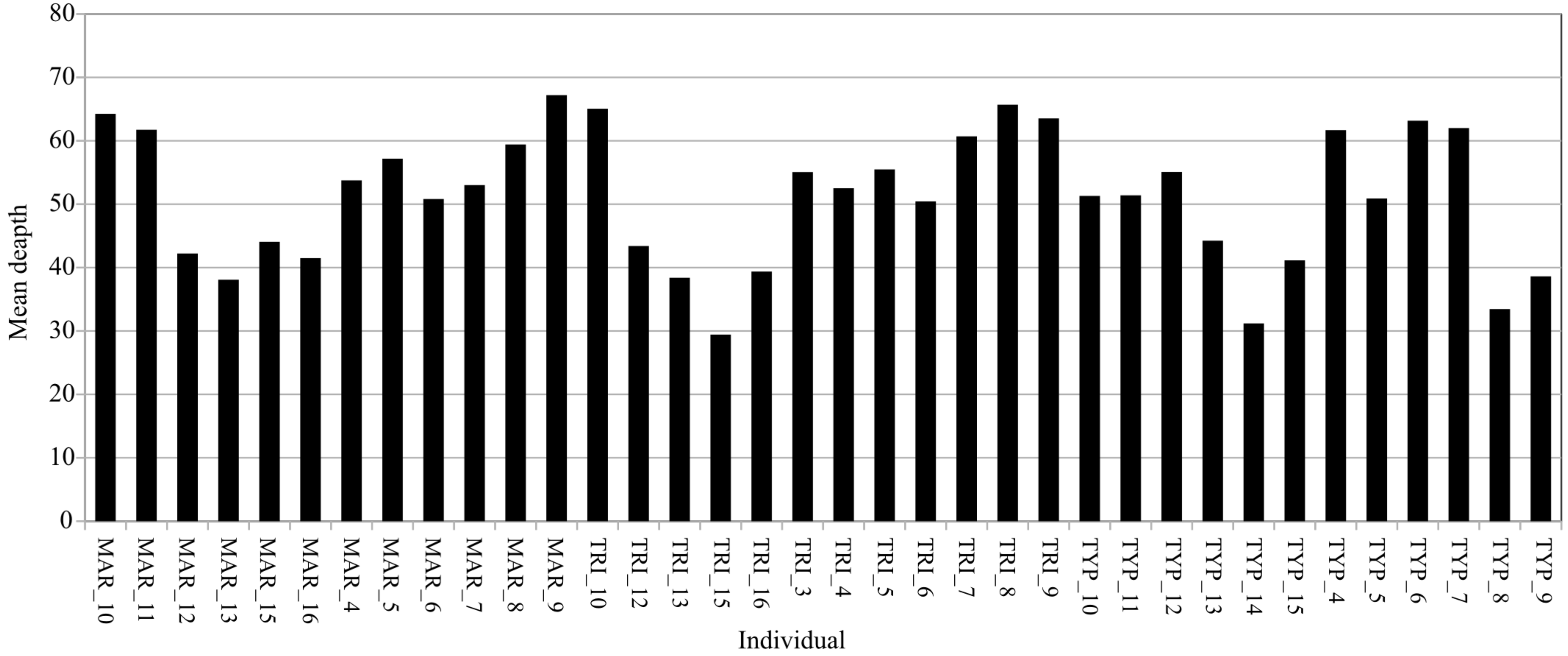

Figure S2 – Mean depth per individual.

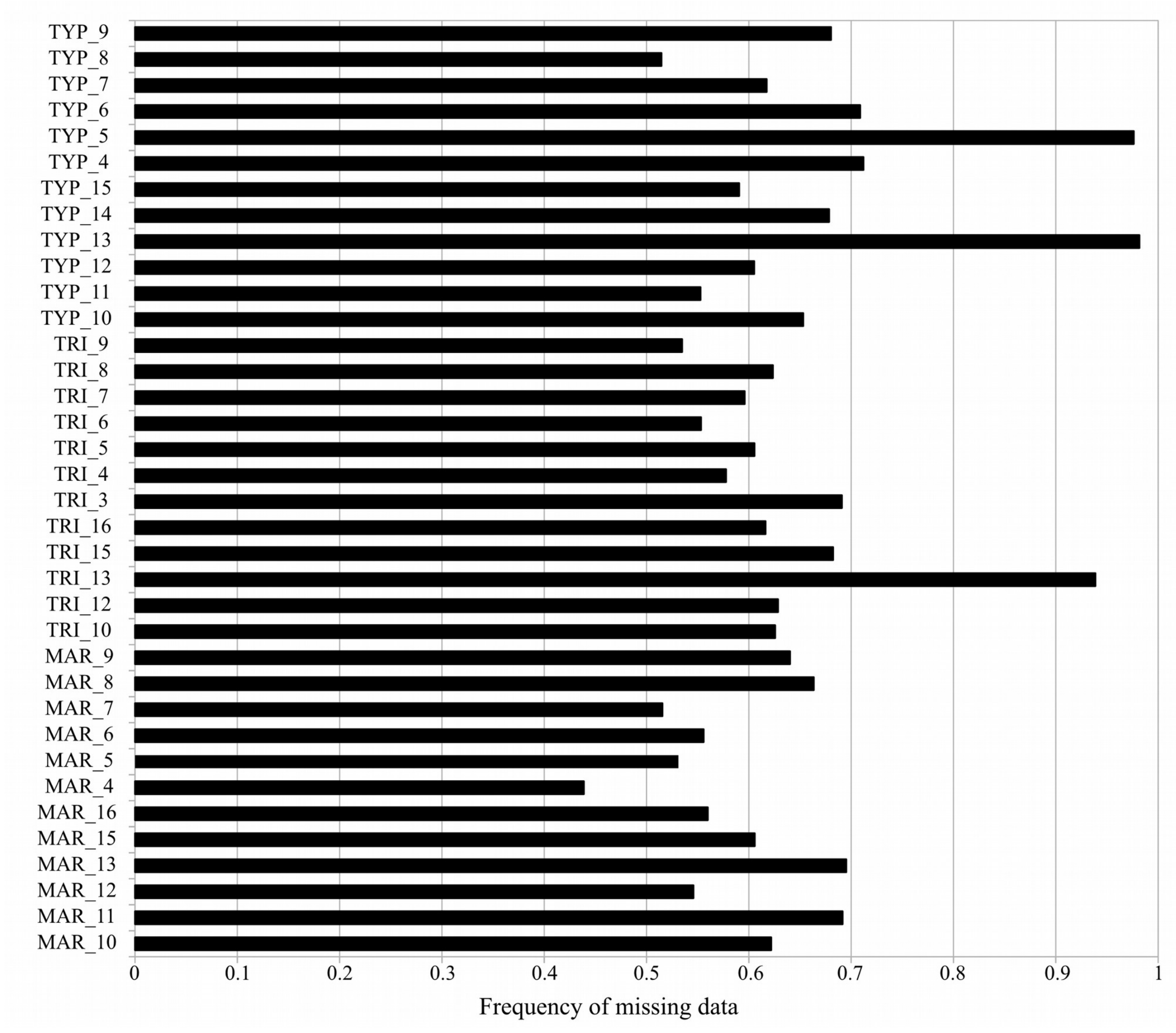

Figure S3 – Proportion of missing data per individual

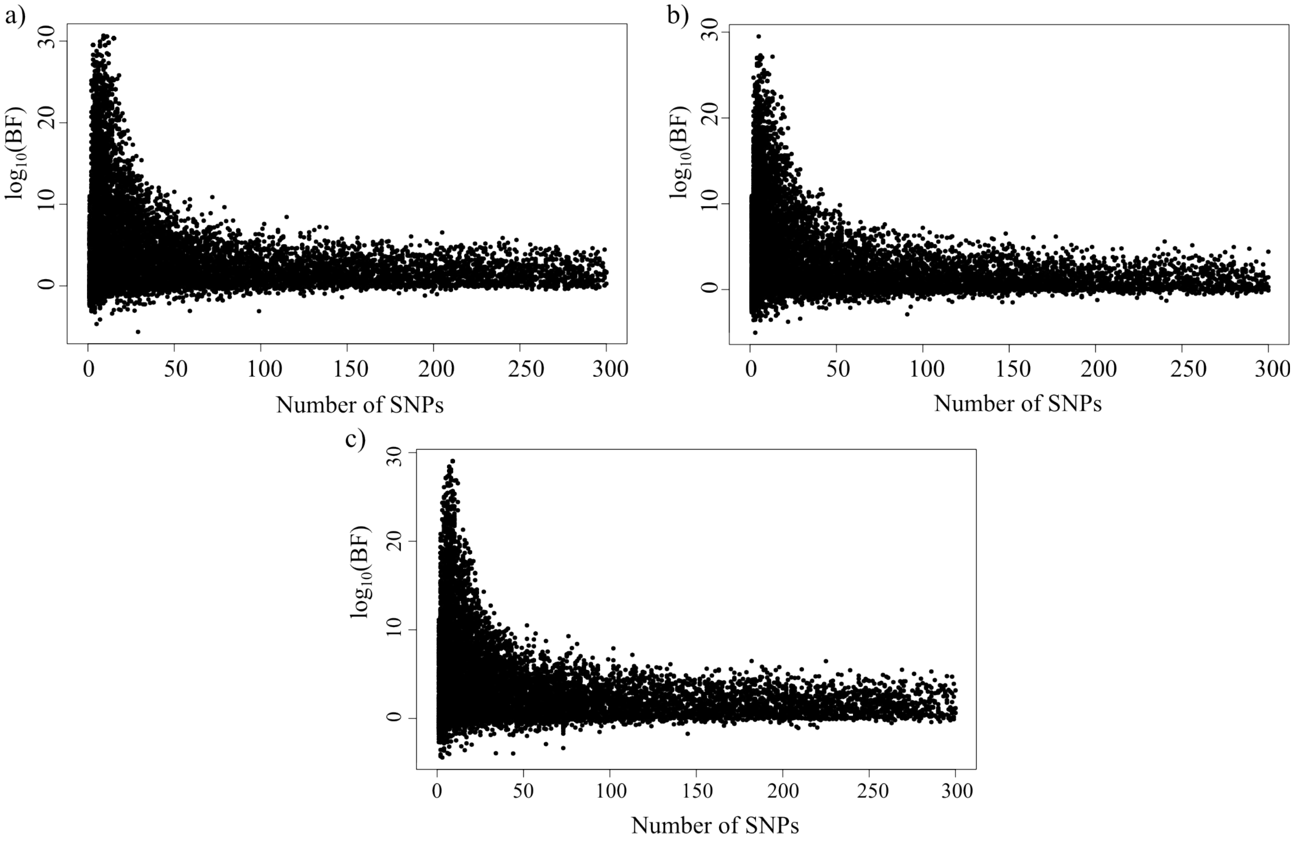

Figure S4 – Number of SNPs in the model as a function of the Bayes factor for each pairwise comparison in multi-SNP association tests. (a) MAR vs TRI; (b) MAR vs TYP; and (c) TRI vs TYP.

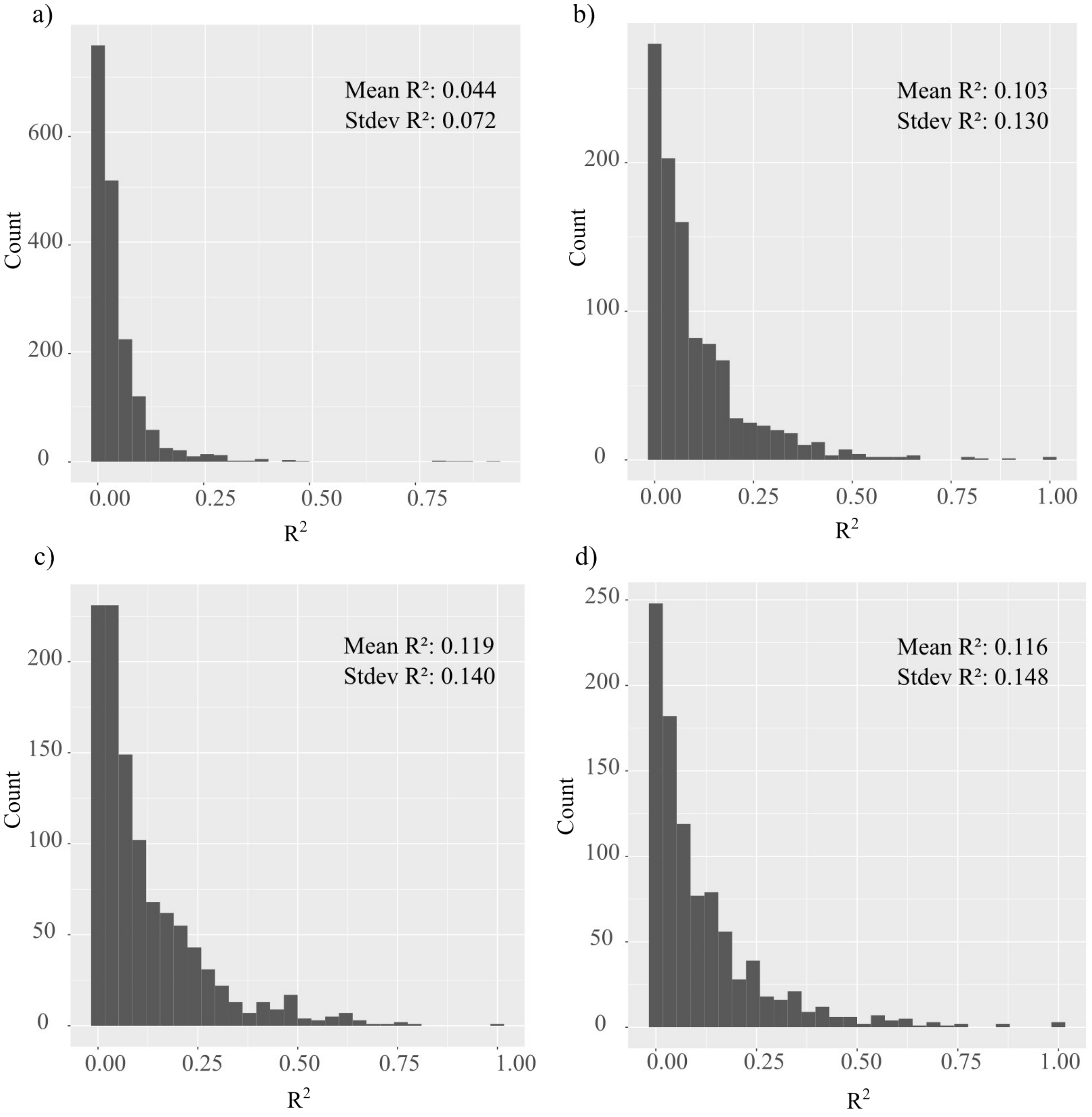

Figure S5 – Histograms with R^2 values for each SNP comparison. Values for the 60 SNPs associated with dorsal colour phenotypes in single and multi-association tests using (a) all individuals; (b) only *marginellus* individuals; (c) only *trilineatus* individuals; and (d) only *typicus* individuals.

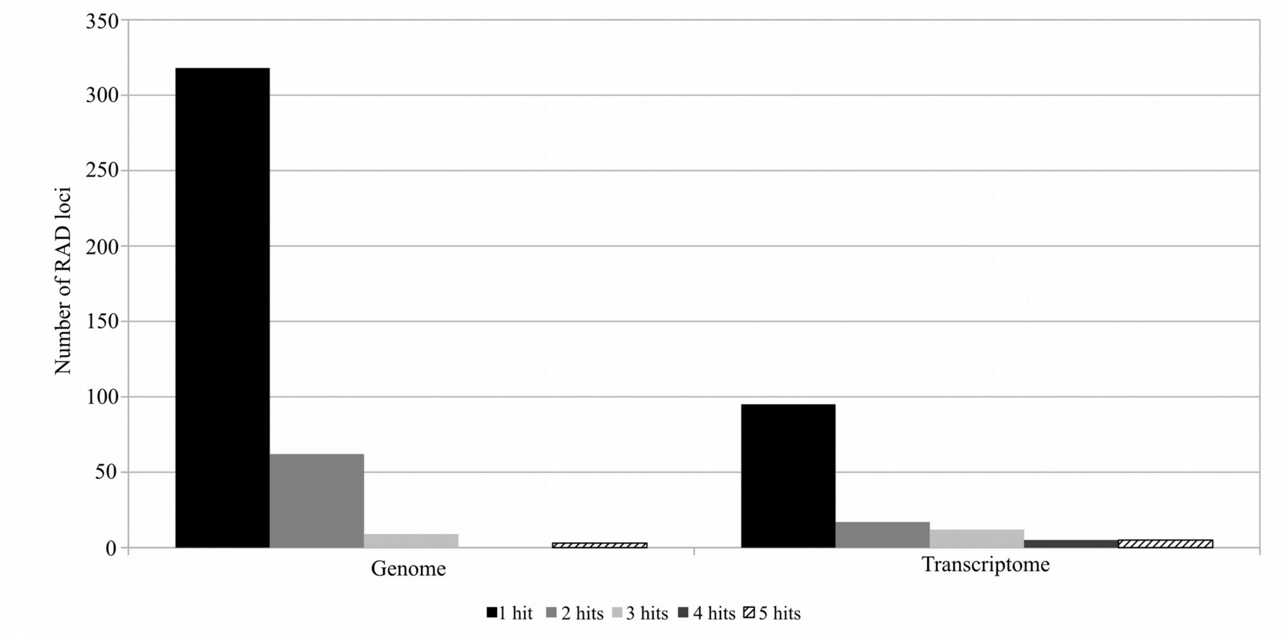

Figure S6 – Number of RAD loci hits with genome and transcriptome contigs/scaffolds.
